# Research Progress and Key Issues of Hydrodebenzylation of Hexabenzylhexaazaisowurtzitane (HBIW) in the Synthesis of High Energy Density Material Hexanitrohexaazaisowurtzitane (HNIW)

**DOI:** 10.3390/ma15020409

**Published:** 2022-01-06

**Authors:** Xiaofei Tang, Rui Zhu, Tianjing Shi, Yu Wang, Xiaochen Niu, Yao Zhang, Junchen Zhu, Wei Li, Wanpeng Hu, Ruoqian Xu

**Affiliations:** 1 Xi’an Modern Chemistry Research Institute, Xi’an 710065, China; tangxiaofei0807@163.com (X.T.); 13488466218@163.com (Y.Z.); z18380461775@163.com (J.Z.); liubliw@163.com (W.L.); 2 College of Biological, Chemical Sciences and Engineering, Jiaxing University, Jiaxing 314001, China; z18324322682@163.com (R.Z.); stj19980802@163.com (T.S.); wangyu180310@163.com (Y.W.); 88888888@zjxu.edu.cn (X.N.)

**Keywords:** hexanitrohexaazaisowurtzitane (HNIW), hexabenzylhexaazaisowurtzitane (HBIW), hydrodebenzylation, catalyst development, catalyst deactivation

## Abstract

High energy density materials (HEDM) are the subject of an extensive research effort in relation to the use of these compounds as components of rocket propellants, powders, and formulations of high-performance explosives. Hexanitrohexaazaisowurtzitane (HNIW, i.e., CL-20) has received much attention in these research fields for its specific impulse, burning rate, ballistics, and detonation velocity. In this paper, the development and performances of the explosives from the first to the fourth generation are briefly summarized, and the synthesis status of the fourth-generation explosive, HNIW, is reviewed. The key issues that restrict the development of industrial amplification synthesis of HNIW are analyzed, and the potential directions of development are proposed. It is pointed out that to synthesize new and efficient catalysts is the key to making the cost-effective manufacturing of CL-20 a reality.

## 1. The Development and Performances of the Explosives

CL-20 (Hexanitrohexaazaisowurtzitane, HNIW) was firstly synthesized at the U.S. Naval Weapons Center in 1987 and became a major breakthrough in the field of high-energy materials. However, the history of explosives can be traced back to the mid-19th century [1].

**Figure 1:**
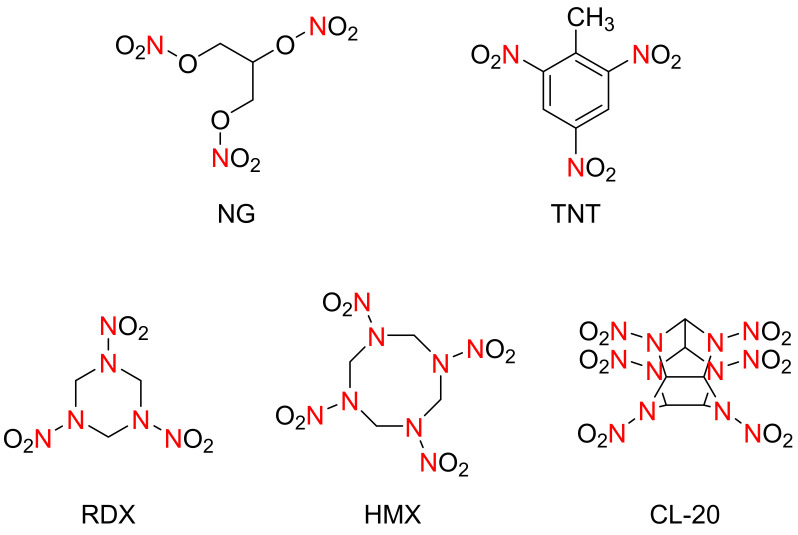
Molecular structure of the I–IV-generation explosives.

When primary explosives, Mercury and Silver fulminates, had been discovered in Europe in the Middle Ages, a whole group of organic nitrates (nitro esters) was synthesized in the first half of the 19th century. For example, Nitrocellulose (pyroxylin) was obtained in 1846 by C. F. Schönbein. Approximately at the same time, Flores Demonte and Ménard received Mannitol hexanitrate (Nitromannite). Nitroglycerin (NG) was discovered by Ascanio Sobrero in 1847, whose molecular structure is shown in Figure 1. During the earlier stage after its discovery, the character of instability limited its application. Aliphatic nitro compounds were also synthesized around the middle of the 19th century. Tetranitromethane, for instance, was synthesized by the Russian chemist L. N. Shishkov in 1857. Trinitrotoluene (TNT), as shown in Figure 1, is a high-synthetic explosive, which was first prepared in 1863 by Julius Wilbrand. Due to its powerful explosive and stable nature, the compound played a huge role in World War II. It was used for the intensive shooting of artillery, and greatly increased the cruelty of the war. Aromatic nitro compounds, such as picric acid (2,4,6-trinitrophenol), were synthesized by Peter Woulfe for the first time using indigo during 1771.

Nitramine, i.e., R1R2N–NO_2_, is an extremely important type of energetic materials [2]. As shown in Figure 1, it mainly includes 1,3,5-trinitro-1,3,5-triazacyclohexane (RDX, found in 1899), and 1,3,5,7-tetranitro-1,3,5,7-tetraazacyclooctane (HMX, found in 1941), and was researched and synthesized after World War II, and had been the most comprehensively applied energetic material up to now. With a detonation velocity of 8500–8600 m/s, the RDX is perfectly suitable for large-scale suppression of multiple-rocket heavy guns, which can greatly improve the power and striking range of the weapon. The detonation velocity of HMX is 9000 m/s, which has a slightly higher impact sensitivity than TNT. It also has good comprehensive performances: it is stable and easier to be detonated. In the Gulf War, it was used for non-contact asymmetric warfare for long-range rocket missiles.

**Figure 2:**
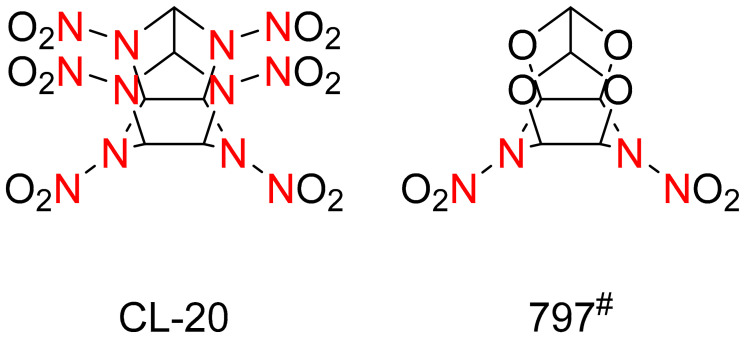
Molecular structures of CL-20 and 797# [4].

In the late 1970s, Yongzhong Yu, a professor at the Beijing Institute of Technology (BIT), and an expert on explosives in China’s “Two bombs and One Star” project, focused on the molecular structure of explosive materials and proposed an innovative theory in which the structure was changed from a two-dimensional flat ring structure to a three-dimensional cage structure [3]. In 1979, Professor Yu and his collaborators successfully synthesized 4,10-dinitro-4,10-diazo-2,6,8,12-tetraoxyisowurtzitane (5.5.0.0^5,9^.0^3,11^) (797^#^, as shown in Figure 2) [4,5], an explosive with a cage structure. They proposed that if the four oxygen atoms in the 797^#^ molecule were replaced by four nitramine groups, the resulting compound, 2,4,6,8,10,12-hexanitro-2,4,6,8,10,12-hexaazaisowurtzitane (5.5.0.0^5,9^.0^3,11^) (HNIW, known as CL-20), would have more comprehensive performances than HMX does, and would become a new-generation HEDM. As can be seen in Figure 2, two five-membered rings connecting four N-NO_2_ groups and a six-membered ring connecting two N-NO_2_ groups together make up the complicated molecular structure of HNIW, where the fist two rings are connected by a C–C bond that has high tension in the polycyclic caged organic compound [1,6]. Professor Yu successfully synthesized this three-dimensional caged structure, CL-20, in 1994 [7]. Since then, a number of experts from BIT began to study the synthesis process of CL-20 [8]. It took 30 years for China to finally solve the key technology of industrial production, from constructing the three-dimensional cage-structure molecules explosive material proposed by Yu Yongzhong, to successful synthesis of 1 kg CL-20 samples using the “One-pot method” suggested by Yuxiang Ou, to the exploration and innovation of process route by Xinqi Zhao, and finally to the breakthrough of the industrial amplification key technology [1,3,9]. The detonation velocity of CL-20 is as high as 9500 m/s, the most powerful non-nuclear elemental explosive [1,3] with respect to being used in practical applications.

**Figure 3:**
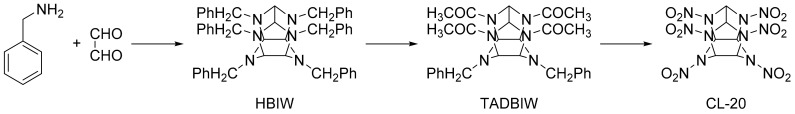
The synthesis route for CL-20 proposed by Nielsen [10].

American scholars proposed the idea of HNIW as a new generation of HEDM research in the early 1980s [9]. During that period of time, Nielsen synthesized HNIW in 1987, and published the synthesis route of CL-20 at the Fraunhofer Institute for Chemical Technology Annual Meeting in Germany in 1996 (see Figure 3). The developed HNIW synthesis route is divided into four processes: the first step is condensation, in which Benzylamine and glyoxal are condensed to hexabenzylhexaazaisowurtzitane (HBIW). The second step is debenzylation, where the six benzyl groups on HBIW are partially or completely converted to acetyl groups or other substituents to form the nitration precursor. The third is nitration, namely, the nitration precursor was nitrated into α-CL-20 or γ-CL-20. The fourth is crystal-structure transformation, which stands for the conversion from α-CL-20 or γ-CL-20 to ε-CL-20. This synthetic route has been widely used up to now. China and the United States independently completed the synthesis of CL-20 on the premise of mutual confidentiality from each other [1].

A comparison of the molecular structures of the fourth-generation explosives is shown in Figure 1, and the physicochemical properties and explosion characteristics of the structures are listed in Table 1 [1].

As can be seen from Table 1, compared with HMX, the “king” of explosives, CL-20 has the advantages of higher energy density, lower oxygen balance, and higher enthalpy of formation [3,7,11]. Therefore, CL-20 has greater potential in applications [12]. For example, it can be used as the most powerful futuristic propellants or explosives due to the higher detonation velocity and detonation pressure. On the other hand, it has the disadvantages of poor sensitivity and low security. These drawbacks have to be fixed before being put into applications.

## 2. Synthesis of HNIW

A great deal of work has been done on the synthesis of CL-20 internationally, concentrating mainly on the following two aspects: the optimization and engineering amplification of the traditional synthesis route proposed by Nielsen, as shown in Figure 3, and the exploration of the new synthesis route of CL-20.

### 2.1. Typical Synthesis Method

Currently, researchers primarily use the route developed by Nielsen to synthesize HNIW (shown in Figure 3); that is, benzylamine (or substituted benzyl) and glyoxal were used as raw materials to produce HBIW through a condensation reaction. HBIW is extremely unstable in nitration medium, which leads to its cage structure opening rapidly, and thus, HNIW cannot be prepared by directly converting the benzyl group (substituted benzyl group) on HBIW to a nitro group. It is obliged to first convert part or all of the benzyl group into other functional groups that must have good stability to this cage structure and that are easy to be converted by the nitro group. Thus, the key step in HNIW synthesis is the debenzylation of HBIW; therefore, the current work introduces the debenzylation process of HBIW in detail [12].

In the HBIW debenzylation process, part or all of the benzyl groups are replaced by other functional groups (such as CH_3_O-, C_2_H_5_-, CHO-) [13]. There are more than 15 nitrification precursors for the synthesis of CL-20 [14,15]. Up to now, only three nitration precursors have been manufactured at the kilogram level, which, respectively, are tetraacetyldibenzylhex-aazaisowurtzitane (TADBIW), tetraacetyldif-ormylhexazoisowoody (TADFIW), and tetraacetylhexazoisowoody alkane (TAIW). The TADBIW system is synthesized from HBIW by hydrodebenzylation once, and the latter two are synthesized from HBIW by hydrodebenzylation twice.

#### 2.1.1. Synthesis of TADBIW

**Figure 4:**
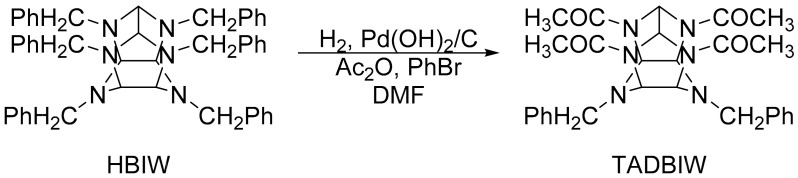
Synthesis of TADBIW.

The most challenging work in synthesizing CL-20 is to convert HBIW to TADBIW (shown in Figure 4), which is caused not only by the multiplex chemical reaction program of HBIW, but also by its unique characteristics [16]. This process is simultaneous debenzylation-acetylation, where the C–N bonds are broken down on the catalysts, which, in turn, causes the forming amines to acetylate with acetic anhydride [16]. Usually, HBIW, dimethylformamide, bromide, and a catalyst containing Pd(OH)_2_ are added to the reactor, and acetic anhydride follows after the air in the reactor is replaced with nitrogen. Then hydrogen is introduced into the reaction under certain conditions. After the reaction is completed, the purified TADBIW is obtained after being washed by solvent. One method to debenzylation has attained the highest level of popularity in the field, in which H_2_ and heterogeneous Pd-based catalysts are implemented in great amounts under catalytic reduction conditions [16]. Additionally, it is crucial to eliminate the benzyl groups under the conditions with the temperature ranges of 30 °C to 60 °C, and with acetic anhydride as an acylation reagent [16]. The complex cage structure is the main factor contributing to the disadvantage that HBIW is unstable and easy to decompose when heated [16]. In order to maintain the cage structure, it is essential to launch the debenzylation and to transform it in a short time at a low temperature (generally 15–23 °C). It has been proved by plenty of optimization experiments that the better starting temperature falls in the range of 17–19 °C. Thus, in the above-mentioned reaction process, the catalysts play an important role when providing high efficiency at around room temperature [12].

Catalyst development. It has taken several decades for researchers to develop Pd/C catalysts as the key for the hydrogenation debenzylation of HBIW [6]. In 1997, Nielsen and Barbara succeeded in synthesizing TADBIW, and they only used 20 wt.% Pearlman’s Pd/C catalyst and nearly the same amounts of HBIW. Based on their works, scientists have investigated numerous Pd/C catalysts for commerce, which have significantly increased the catalytic activity [6]. In 1997, Wardle revealed that Degussa E101 NE/W had yields reaching 90% for hydrogenating HBIW when keeping the scale in a 50-g process, and with help from bromobenzene. Additionally, the material is recycled once more. Using different carbon precursors as catalysts supports, and subunit-supported 6 or 10 wt.% Pd had a yield as high as 82% when keeping the experiment scale in a 3-g process [17]. It was reported that in the chemical process, ACROS organic active carbon over a Pd catalyst performed better than other types of activated carbons [17]. Disordered mesoporous carbon (MC) had a yield of TADBIW as high as 80%, a very good catalytic reactivity, when it was prepared using the hard template method [6], while the yield of the prepared Pd(OH)_2_/MC catalyst, as a comparison, remained above 70% after two cycles. The researchers found that it is hard to obtain the target product when loading Pd with a concentration lower than 5 wt.% if the usage amount of the catalysts remains constant [17].

Effect factors. It has been revealed by researchers that the catalytic activity of Pd (OH)_2_/C is significantly affected by the following factors [18,19,20,21], such as the surface physical properties of activated carbon, reaction temperature, and the loading amount of Pd, etc. It has also been revealed that lower temperatures make Pd(OH)_2_ particles disperse better. In the 20-kg scale, a series of amplification trials has implemented the catalyst for the hydrogen debenzylation from HBIW, and the results showed that the yield of the TADBIW product reached as high as 82%.

Effects of additives containing bromine. In 1995, Wardle and Edwards [22] found that the addition of bromide made a cost-effective way of synthesizing HNIW. When Pd(OH)_2_ was used as catalyst without support, a small amount of bromobenzene could help receive excellent effects, such as shorter reaction times, less catalyst amounts, and higher yields of TADBIW (from 68.3% to 80%). Weirong Han and Yuxiang Ou et al. [21] also drew a similar conclusion after investigating the effects of 10 types of bromides, including bromobenzene, imposing on HBIW catalytic hydrodebenzylation, and discussed the mechanisms. Lianzhong Chen and Xinqi Zhao et al. [23] found that switching bromobenzene to bromotoluene (o, m, p—mixture) in the primary hydrodebenzylation of HBIW improved the hydrodebenzylation yield, and reduced the amount and toxicity of bromine-source substances. In addition, Weirong Han [24,25] and Yuxiang Ou et al. [26,27] prepared triacetyltribenzylhexazazoisovudane (TATBIW), an important intermediate in the hydrolytic reaction, analyzed its single-crystal structure (TATBIW·0.5H_2_O) [28], and discussed the mechanism and reaction kinetics of the reaction.

Process development. Professor Yongzhong Yu, respectively, synthesized TADBIW in 1993 and CL-20 [4] in June 1994, which coincides with the later-reported methods proposed by Nielsen, France, etc. In 1995, Bellamy published a research report [29] on the preparation of TADBIW from HBIW by hydrodebenzylation-acetylation, pointing out that the yield of the product had been improved by appropriately increasing the reaction temperature, prolonging the reaction time, or increasing the amount of catalyst, i.e., Pd(OH)_2_/C. It concluded that direct nitration TADBIW produced high-yield CL-20, but the specific nitration medium and related process conditions were not provided [6,29]. Since TADBIW can be obtained by hydrodebenzylation only once, many countries and institutes initially adopted the one-pot nitration route for the synthesis of CL-20 [30,31,32,33], which is easy to realize at the laboratory scale. Some countries, such as the United States and France, have found that this method is not suitable for engineering amplification [34,35], because it takes a large amount of noble metals and is easy to inactivate, resulting in a high cost [36], while the existing economical catalysts are not recyclable, limiting their mass production and comprehensive application [16]. However, other countries, such as Iran and Russia, are still continuing the relevant research [37,38,39,40].

#### 2.1.2. Synthesis of TADFIW

**Figure 5:**
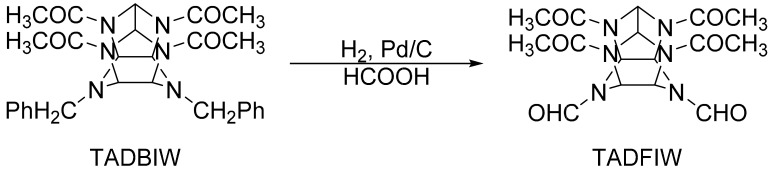
Synthesis of TADFIW.

As shown in Figure 5, the synthesis of TADFIW is based on TADBIW. Add TADBIW and formic acid into a reaction bottle, remove the air in the reaction bottle, and introduce normal-pressure hydrogen at a certain temperature for hydrogenolysis. The reaction stops when the system no longer absorbs hydrogen. After the reaction is finished, the TADFIW with certain purity can be obtained after the operations of washing, decompression, and the like. In 1997, Thiokol published the detailed process and conditions for the synthesis of HNIW by using TADFIW as a nitration precursor. In this process, bromobenzene was used as the bromine source and dimethylformamide (DMF) as the solvent [22,41]. DMF is a Lewis base, which can neutralize the acid generated in the medium or in the reaction process, keep the medium in a low-acidic environment, increase the solubility of HBIW in the hydrolytic medium, accelerate the reaction speed of hydrolytic acetylation, reduce the existence time of HBIW in the reaction medium, prevent and halt the breakdown of the cage structure, and reduce the decomposition of the reaction substrate. The yield of TADBIW reached more than 80% by Thiokol, and it was further hydrolyzed in HCOOH medium to obtain TADFIW. TADFIW was then nitrated in nitric-sulfuric mixed acids to form the target product, CL-20, with a high yield. The United States used the TADFIW route to prepare CL-20 up to ten tons. However, formyl acetyl was difficult to be separated, which resulted in the CL-20 containing 2%~3% of pentanitromonoformylhexazazoisowoodane impurity, which affected the detonation and the safety performance of CL-20 [3,42,43]. After 2002, the United States abandoned the TADFIW route; however, some countries, such as Iran, are still working on the relevant work [20].

#### 2.1.3. Synthesis of TAIW

**Figure 6:**
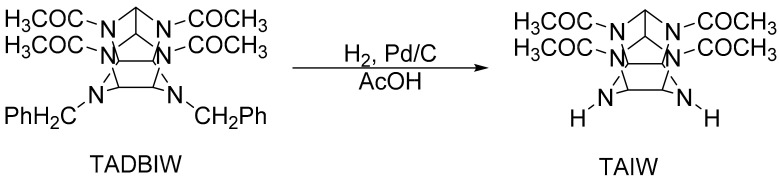
Synthesis of TAIW.

HAIW is an excellent intermediate for the synthesis of HNIW. The synthetic route is as follows: TADBIW is placed in a mixed solution of methanol and formic acid to form a slurry. Add Pd/C catalyst into that slurry under stirring, heat it for a certain time, and then filter and extract it to separate the TAIW from it. This route was pioneered in China. In 1994, Xinqi Zhao hydrogenated TADBIW in acetic acid medium to prepare TAIW, and then synthesized CL-20 from TAIW [44]. Cai Wang and Yuxiang Ou et al. [45,46] studied and reported the crystal structure of HAIW. As shown in Figure 6, Jinquan Liu and Yuxiang Ou et al. used propionic acid and n-butyric acid as hydrogenolysis media, and a self-made Pd substrate as a catalyst to hydrolyze TADBIW for producing TAIW [47]. The hydrodebenzylation of TADBIW in HCOOH/CH_3_OH medium for obtaining HAIW was studied by Thiokol [22,41].

As the intermediate production, TADBIW showed higher performance than HBIW in terms of stability and durability at high temperatures. To accelerate the process of the debenzylation of TADBIW, the heating can be maintained at higher temperatures (usually 34–40 °C) [6]. It is difficult for TADBIW to solutate in other alternative solvents; its debenzylation must be conducted in formic or acetic acid solution. However, the catalysts in the acid solvents can be etched by the solution, resulting in poor cycle catalytic properties, which has been reported. There is a declining trend in the catalytic performances for the hydrogenation of N,N-dibenzyl-3-phenylindolizin-1-amine when it is in the acetic acid solution with cycles over recycled PtO_2_ and Pt/C [12]. According to the report, the catalysts must be stable so that they can resist the acid corrosion. The Pd/C with 10% loading is the most common material in the field for the hydrodebenzylation of HBIW and TADBIW. However, the low atom-utilization rate, as well as the loss and aggregation of Pd, restrain the catalytic performance. In the field of HEDMs, it is a significant work to develop a new catalyst that has high effectiveness for the debenzylation of both HBIW and TADBIW [12].

At present, most countries have adopted the TAIW route for synthesizing CL-20 [37,48,49]. The nitration of TAIW for producing high-purity CL-20 has been achieved smoothly by using industrial nitration agents in standard nitration units [43]. The TAIW route requires noble metal Pd-based catalysts for hydrodebenzylation twice; thus, the preparation of an efficient Pd catalyst and the recycling of Pd catalysts are the key technologies to make CL-20 for wide use [7,12].

### 2.2. Other Synthesis Methods

#### 2.2.1. Non-Hydrodebenzylation-Oxidation Debenzylation of HBIW

Due to the long process route of HBIW hydrodebenzylation and nitration, and the massive requirement of noble metal catalysts in the hydro removal of benzyl groups, the manufacture of CL-20 costs huge sums of investment in reality. In order to reduce the cost and simplify the synthesis procedures of CL-20, researchers all over the world are trying to develop new synthetic routes [7].

HBIW reacts with trimethylsilane ethyl chloroformate, creating the corresponding substituted products in solvent-mixed tetrahydrofuran and ethyl ether, which has been revealed by the Asahi Kasei Corporation. This reaction requires chloroformate-substituted products which can be generated in an inert gas environment, and CL-20, which is obtained when there is nitrite and nitric acid. Because chloroformate is highly toxic and expensive, the synthesis cost of CL-20 by this method is much higher than the hydrodebenzylation method; therefore, this method has not been applied in practice. Surapaneni et al. [50], from the US Army Research and Development Center, failed to achieve the hydrogen-free debenzylation of HBIW using strong Lewis acid, because HBIW decomposed easily under the reaction conditions. Siping Pang and Yongzhong Yu from BIT studied the oxidation debenzylation reaction of HBIW or HADBIW [51,52,53,54,55,56,57,58,59,60,61,62], and made the oxidation debenzylation manufacturing of CL-20 a reality, using KMnO_4_ and ammonium nitrate (IV) as oxidants, CH_2_Cl_2_ and DMF as solvents, Ac_2_O and nitric acid (65%)/sodium nitrite as reactants, and the combination of boron trifluoride ether, ammonium persulfate, sodium nitrite, anhydrous sodium carbonate, and tetraethylammonium bromide as catalysts. Since then, similar studies have been carried out in India and other countries [63]. Due to plenty side-reactions, the yield and purity of the products by this method cannot meet the requirements of engineering production, so it does not have practical application [7]. The bottleneck of a hydrogen-free solution lies in the poor stability of HBIW in the reaction medium, and the reaction activity of the six benzyl groups in HBIW is different, which is difficult to be completely converted at once [7].

#### 2.2.2. Non-HBIW Route—Synthesis of Other Iso-Woodsane Precursors

The non-HBIW route attempts to synthesize other iso-woodsane precursors and then direct nitrate into CL-20, aiming to shorten the synthesis route and reduce the cost. Sysolyatin et al. [64], from Russia, reported the condensation of sulfamate with glyoxal to obtain the caged precursor of isodurane and the product nitrated into CL-20. In 2005, Cagnon et al., from a French dynamite company [65], used aromatic heterocyclic methylamine or allylamine with acetate glyoxal to synthesize hexaazazoisovaltane, which was then directly nitrated to obtain CL-20. On the basis of the above-mentioned methods, Chapman et al. [66,67], from the United States, used photocatalytic and oxidation methods to make the isomerization of hexaallyl hexaazazozygrotane, and nitrated the product to CL-20. Related studies have also been carried out in Poland and other countries, but the researchers did not make much substantial progress. The researchers from BIT [68,69] also synthesized some new isowurtzitane derivatives. Moreover, Anxin Hou, from the Southeast University of China [70], synthesized a cage precursor, hexafurfurylhexaazaisowurtzitane (HFIW), and directly nitrated the HFIW into HNIW. All above works have the problems of low yield of HNIW and no breakthrough in the practical application of engineering.

#### 2.2.3. High-Energy Materials with Non-Cage Structures—Three-Dimensional MOFs with Nitrogen-Rich Elements

In order to resolve the contradiction between high energy and high sensitivity in the development of energy materials, Siping Pang et al., from BIT [1], designed and synthesized nitrogen-rich three-dimensional MOFs. The presence of nitrogen-containing heterocycle ligands guarantees the synthesized material with high energy density, while the sensitive nitrogen and metal atoms are clad in the three-dimensional structure, which ensures the decrease of sensitivity and the improvement of safety.

The above new synthesis technologies of CL-20 have encountered the bottleneck of low yields and side-reactions, and the characteristics of the cage structure of isodurane was unstable and easy to be destroyed, which makes the mild catalytic hydrodebenzylationbe an inevitable choice. To break through the bottleneck, there are needs for new methods, or new technologies. However, it is not a short-term work; thus, the HBIW hydrodebenzylation route is still a feasible means for mass production [7].

## 3. Problems in HNIW Engineering Manufacturing

Many countries have carried out a lot of works on the synthesis and manufacture of CL-20. Although the engineered CL-20 has been made into a reality, and though the cost has been gradually reduced, it is still relatively high compared to the third-generation energetic compounds RDX and HMX. Practices have proved that only the hydrogenolysis debenzylation method, using precious metal Pd as a catalyst, has practical significance.

At present, researchers have recognized [18,19,20,21] that a carbon-supported Pd catalyst is the most effective catalyst for HBIW hydrodebenzylation reaction, and the amount of Pd is about 20 wt.% of the supporting carbon-based material. When the amount of Pd loaded on the catalyst was 0.3 wt.% of the substrate HBIW, the hydrogenolysis debenzylation acetylation product yielded up to 93%. Weirong Han and Yuxiang Ou et al. [71] found that the yield of TADBIW could reach 88% when the amount of Pd was 0.2 wt.% of HBIW. The carbon-based material-supported Pd catalysts have high conversion rates and selectivity, but the reaction activity of the Pd-catalyzed hydrodebenzylation process is unstable; sometimes the catalytic effect significantly declined, and even catalyst poisoning can occur, which results in hydrodebenzylation failure and the difficulty to reuse catalysts [65]. All the above reasons make HNIW synthesis price too high, and is the biggest obstacle to mass production of the energetic material HNIW.

Wardle [22] found that N-benzylacetamide, the by-product in the HBIW hydrodebenzylation process, led to Pd catalyst poisoning, and he also found that the generation of benzylamine was controlled by regulating the temperature of the hydrodebenzylation reaction. However, it is inevitable to contain a certain amount of N-benzylacetamide in HBIW during its mass-production process. Its purity can reach higher than 98% after being refined through recrystallization, but it still contains a small amount of impure oxalodibenzylamine produced in the oxidation of aldehyde amine condensation intermediates, C_6_H_5_CH_2_NHCH(OH)CH(OH)NHCH_2_C_6_H_5_, and affects the performance of the Pd-based catalyst during the hydrodebenzylation process [72,73]. I. L. Simakova et al. [39] discovered that the rapid deactivation of a Pd/C catalyst in the HBIW hydrodebenzylation process was not determined by the preparation method, but the agglomeration of metal particles and the blocking of activated carbon particles by oligomer products inside the carbon pores. Hadis Bashiri et al. [74] found that the loading amount of Pd decreased in the HBIW hydrodebenzylation process, and believed that the leaching and aggregation of Pd particles was responsible for the deactivation of the catalyst. To reduce the loading amount of Pd and improve the stability of the Pd-based catalyst, JunYang and Shuang Liu, from the Shanghai Institute of Organic Chemistry, Chinese Academy of Sciences, studied [11,12,16] the application of bimetallic catalysts in HBIW hydrodebenzylation reactions, and found that when the amount of the catalyst was the same, the yield of a bimetallic catalyst (PdFe) was significantly higher than that of the Pd mono-metal catalyst, and was 4% higher than that of the commercial Pd/C catalyst.

Although there have been reports on the deactivation reasons for HBIW hydrodebenzylation reaction carbon-supported Pd catalysts and the design and development of new catalysts [1,6], these studies are rarely systematic and are not published. In order to save the cost and further reduce the consumption of raw materials, in-depth research on the causes and mechanisms of catalyst deactivation play a great guiding role in the optimization of the catalyst structure, the construction of new catalyst structures, and the improvement of reaction processes. In the HBIW hydrodebenzylation process, under the precondition of maintaining the catalytic activity and selectivity of HBIW for hydrodebenzylation reaction, seeking cheap and efficient new catalysts, reducing the use of palladium, or prolonging the life of Pd-based catalysts are the development directions for eventually making the large-scale production and application of HNIW a reality.

## Figures and Tables

**Table 1:** Comparisons of the physicochemical properties and explosion characteristics of the II–IV-generation explosives [1,10].

ITEM	TNT	RDX	HMX	CL-20
Density/g·cm^−3^	1.65	1.82	1.90	2.04
Relative density/g·cm^−3^	91.64	91.77	91.88	-
Oxygen balance/%	−74.0	−21.6	−21.6	−10.95
Standard enthalpy of formation/kJ·mol^−1^	−45.4	92.6	104.8	416.28
Detonation velocity/km·s^−1^	6.9	8.6	9.0	9.6 **
Detonation pressure/GPa	19	34	39	43 **
Critical capacity/cm^3^·g^−1^	738	903	886	827 **
Friction sensitivity */N	353	120	120	54
Impact sensitivity/N·m	15	7.4	7.4	4
Flash point/°C	300	230	287	228

* The smaller the value, the higher the friction sensitivity; ** Calculated value.

## Data Availability

Not applicable.

## Nomenclature

HBIW	hexabenzylhexaazaisowurtzitane
HFIW	hexafurfurylhexaazaisowurtzitane
HAIW	hexaacetylhexaazaisowurtzitane
HEDM	high energy density materials
HNIW	hexanitrohexaazaisowurtzitane
TADBIW	tetraacetyldibenzylhex-aazaisowurtzitane
TADFIW	tetraacetyldif-ormylhexazoisowoody
TAIW	tetraacetylhexazoisowoody
NG	nitroglycerine
TNT	trinitrotoluene
RDX	1,3,5-trinitroperhydro-1,3,5-triazine
HMX	octahydro-1,3,5,7-tetranitro-1,3,5,7-tetrazocine
